# DNA hypermethylation driven by *DNMT1* and *DNMT3A* favors tumor immune escape contributing to the aggressiveness of adrenocortical carcinoma

**DOI:** 10.1186/s13148-023-01534-5

**Published:** 2023-08-02

**Authors:** Gwenneg Kerdivel, Floriane Amrouche, Marie-Ange Calmejane, Floriane Carallis, Juliette Hamroune, Constanze Hantel, Jérôme Bertherat, Guillaume Assié, Valentina Boeva

**Affiliations:** 1 INSERM, U1016, Cochin Institute, CNRS UMR8104, University of Paris, 24 rue du Faubourg Saint-Jacques, Paris, France; 2 Inovarion, Paris, France; 3 Department of Endocrinology, Diabetology and Clinical Nutrition, University Hospital Zurich (USZ) and University of Zurich (UZH), Zurich, Switzerland; 4 Medizinische Klinik und Poliklinik III, University Hospital Carl Gustav Carus Dresden, Dresden, Germany; 5 Department of Computer Science, Institute for Machine Learning, ETH Zurich, Universitätstrasse 6, 8092 Zurich, Switzerland; 6 Swiss Institute of Bioinformatics (SIB), Zurich, Switzerland

**Keywords:** DNA methylation, CIMP, DNA methyltransferases, DNMTs, Demethylating agents, 5-azacytidine, Adrenocortical carcinoma, Immune escape

## Abstract

**Background:**

Adrenocortical carcinoma is rare and aggressive endocrine cancer of the adrenal gland. Within adrenocortical carcinoma, a recently described subtype characterized by a CpG island methylator phenotype (CIMP) has been associated with an especially poor prognosis. However, the drivers of CIMP remain unknown. Furthermore, the functional relation between CIMP and poor clinical outcomes of patients with adrenocortical carcinoma stays elusive.

**Results:**

Here, we show that CIMP in adrenocortical carcinoma is linked to the increased expression of DNA methyltransferases *DNMT1* and *DNMT3A* driven by a gain of gene copy number and cell hyperproliferation. Importantly, we demonstrate that CIMP contributes to tumor aggressiveness by favoring tumor immune escape. This effect could be at least partially reversed by treatment with the demethylating agent 5-azacytidine.

**Conclusions:**

In sum, our findings suggest that co-treatment with demethylating agents might enhance the efficacy of immunotherapy and could represent a novel therapeutic approach for patients with high CIMP adrenocortical carcinoma.

**Supplementary Information:**

The online version contains supplementary material available at 10.1186/s13148-023-01534-5.

## Introduction

Adrenocortical carcinoma (ACC) is an endocrine cancer of the adrenal gland with an annual incidence of approximately 1 per million [1]. Albeit rare, ACC is extremely aggressive characterized by inter-patient heterogeneity and 16–44% five-year survival rate [2, 3]. Complete surgical removal of localized tumors represents the only curative option; nevertheless, the recurrence rate remains high (30–75%) [1, 4]. Alternatively, unresectable or metastatic ACCs receive mainly palliative treatments including the adrenolytic drug mitotane, chemotherapy, and radiotherapy [5].

Substantial recent efforts focused on obtaining a comprehensive genomic characterization of ACC, including The Cancer Genome Atlas (TCGA) Research Network [6] and the European Network for the Study of Adrenal Tumors (ENSAT) [7], have provided integrated analysis of multidimensional genomic data to better predict patient outcomes and help develop new therapeutic approaches. Genes identified as potential drivers of ACC tumorigenesis include insulin-like growth factor 2 (highly expressed in approximately 80% of ACC), as well as β-catenin, *TP53*, *ZNRF3*, *CDKN2A*, *RB1*, *MEN1*, *DAXX*, *MED12* and *TERT* (mutated in, respectively, 16, 16, 21, 11, 7, 7, 6, 5, and 6% of ACC) [6, 7]. However, about 30% of ACC cases lack such mutations [7]. Additional factors associated with increased mortality include copy number alterations, gene expression changes, and aberrant DNA methylation; specifically, the CpG island methylator phenotype (CIMP).

CIMP, first described in colorectal carcinoma [8], is characterized by hypermethylation of hundreds of CpG islands surrounding gene promoter regions. CIMP is observed in numerous cancer types [8–11] with differing effects on prognosis. In various cancers, but not in ACC, the contribution of DNA hypermethylation to cancer progression and aggressiveness has been linked to the immune escape [12–15]. Specifically, treatment with demethylating agents has been shown to improve the survival of the patients with such cancers, and to increase the efficiency of immunotherapy [16]. However, despite the fact that CIMP status is used as a biomarker of aggressiveness in ACC, we still do not know what drives the poor survival of CIMP patients and whether their poor survival is connected to the immune escape in this particular cancer type.

Moreover, the mechanisms underlying CIMP establishment in ACC are unclear [17]. Particularly, no association of CIMP with any specific mutation has been observed neither in the ENSAT cohort nor in the TCGA cohort [6, 7].

In this study, we identified potential molecular drivers of DNA hypermethylation in ACC and demonstrated its importance in ACC progression and outcome. Our results provide new insights into the role of DNA methylation in ACC tumorigenicity and suggest that DNA demethylating agents may be beneficial for patients with CIMP, particularly in association with immunotherapy.

## Results

### CIMP in ACC is associated with increased *DNMT1* and *DNMT3A* expression

**Fig. 1:**
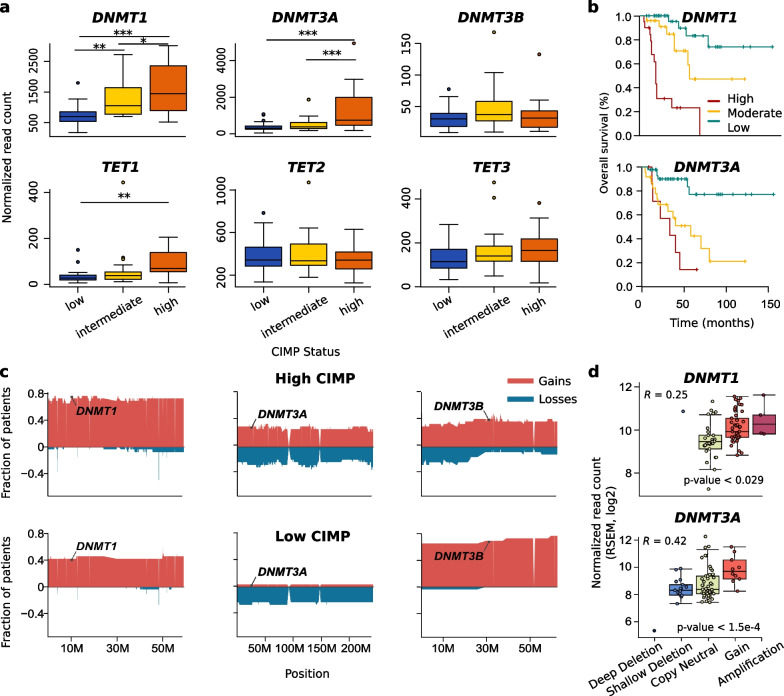
*DNMT1* and *DNMT3A* expression levels are augmented in hCIMP ACC and associated with increased copy numbers. **a** Expression levels of *DNMT1*, *DNMT3A*, *DNMT3B*, *TET1*, *TET2*, and *TET3* in TCGA patients exhibiting lCIMP (*n* = 32), iCIMP (*n* = 26), and hCIMP (*n* = 21) (two-sided *t* test, ***p < 0.005). **b** Kaplan–Meier estimates of overall survival for ACC patients, as a function of *DNMT1* or *DNMT3A* expression (divided as high, medium and low) for the TCGA dataset. **c** Pattern of copy number alterations in hCIMP and lCIMP ACC in chromosomes containing *DNMT1*, *DNMT3A*, and *DNMT3B* genes. **d** Expression levels of *DNMT1* and *DNMT3A* in TCGA patients according to copy number status of the genes

To determine factors that are associated with and might contribute to the establishment of CIMP in ACC, we analyzed publicly available microarray and RNA-Seq data from ENSAT and TCGA [6, 7]. We focused on the expression levels of genes coding for factors involved in DNA methylation or demethylation, which were derived from the EpiFactors database [18], as a function of the CIMP status (Additional file 2: Table S1). ACC samples were characterized as low, intermediate, or high CIMP (lCIMP, iCIMP, hCIMP) as described in Assié et al. [7] and Zheng et al. [6] for ENSAT and TCGA cohorts, respectively. Among all genes known to be positively related to DNA methylation [18], only DNA methyltransferases *DNMT1* and *DNMT3A* exhibited significantly higher expression in hCIMP than lCIMP cases in both TCGA and ENSAT datasets with minimal expression fold change above 1.4 (Fig. 1a, Additional file 1: Fig. S1, Additional file 2: Table S1, TCGA cohort *p* values 9.23e−07, 7.28e−06 and ENSAT cohort *p* values 1.62e−04 and 1.23e−02 for *DNMT1* and *DNMT3A* expression changes, respectively). This suggested that increased expression of these two factors may contribute to the establishment of the CIMP phenotype. In line, high expression of these factors was also associated with a poor overall survival (TCGA dataset: *DNMT1* Bonferroni adjusted *p* value < 5.16 × 10^−5^ and *DNMT3A* p value < 6 × 10^−5^; ENSAT dataset: *DNMT1* *p* value < 1.22 × 10^−5^ and *DNMT3A* *p* value < 0.146; Fig. 1b, Additional file 1: Fig. S1). Additionally, expression of *TET1* and to a lesser extent *TET3*, which code for enzymes involved in DNA demethylation, was also increased in hCIMP compared to lCIMP tumors. However, the expression level of these genes remained low compared to that of *DNMT1* and *DNMT3A* (Fig. 1a, Additional file 1: Fig. S1).

### Copy number aberrations and increased proliferation together contribute to higher DNMT expression in ACC

To clarify the causes underlying *DNMT1* and *DNMT3A* overexpression in ACC, we analyzed copy number alterations in the *DNMT* loci (Fig. 1c). The frequencies of gains in *DNMT1* and *DNMT3A* were increased in hCIMP tumors compared to those in lCIMP, whereas fewer *DNMT3B* gains were observed in hCIMP. Further, *DNMT1* and *DNMT3A* expression levels significantly correlated with copy number alterations, the Pearson correlation coefficients (*R*) were 0.25 and 0.42, respectively (Fig. 1d). However, as it could be seen from the correlations, copy number status did not fully explain the augmented expression of DNMTs, which made us search for further drivers of increased DNMT expression in the hCIMP ACC tumors.

We hypothesized that increased cell proliferation observed in aggressive ACC tumors presenting CIMP [6] can contribute to the augmented expression of DNA methyltransferases, independently from the gene copy number status (Pearson’s correlation on TCGA ACC samples between the CIMP score and proliferation score: *R* = 0.51, *p* = 1.5e−06; between the CIMP score and mitoses per 50 high-power field: *R* = 0.58, *p* = 3.84e−06). Indeed, *DNMT1* and *DNMT3A* expression is elevated during S phase in order to prepare the reproduction of the methylation profile in the daughter cells [19]. Also, in prostate cancer and acute myeloid leukemia, DNA hypermethylation has been shown to be a consequence of hyperproliferation [20, 21]. To check this hypothesis in ACC, we compared sample *DNMT1* and *DNMT3A* expression levels with the proliferative status. Because the mitotic rate was not available for all tumors, we computed a proliferative score using ROMA [22]. The correlation between proliferation score and mitotic rate was high and significant (Spearman *ρ* = 0.522, *p* value < 5.24e−5, *n* = 54); the deviation from perfect correlation is likely to be explained by facts that the mitotic rate was counted manually and a proliferation score based on RNA-seq measurements was calculated from a slightly different part of the tumor.

**Fig. 2:**
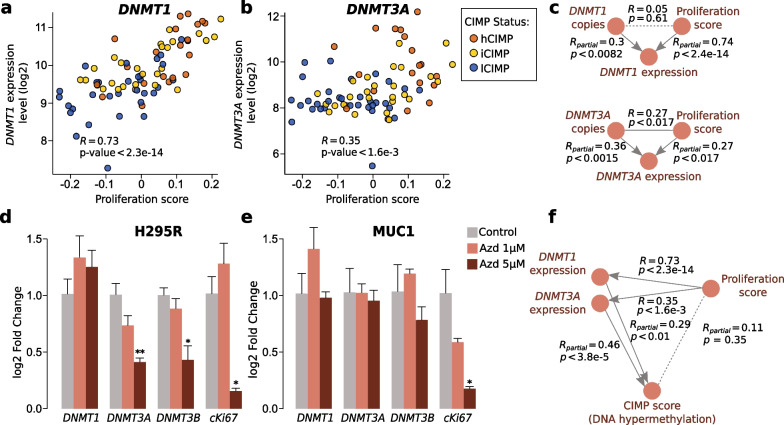
Increased cell proliferation in hCIMP ACC tumors is a likely second driver of augmented *DNMT1* and *DNMT3A* expression, along with copy number aberrations in the *DNMT* genes. **a** and **b** Correlation between *DNMT1* (a) or *DNMT3A* (b) expression and cell proliferation; hCIMP samples (*n* = 21, orange), iCIMP (*n* = 26, yellow), and lCIMP (*n* = 32, blue). **c** Modeling of inter-dependencies between genomic copy number status of *DNMT* genes, cell proliferation and gene expression using partial correlations for the TCGA data set. *R*_partial_ stands for the Pearson partial correlation coefficient calculated when controlling for the third factor. **d** and **e** Effect of a 48 h treatment with the proliferation inhibitor AZD-5438 (Azd) at 1 or 5 µM on the expression of *DNMT1*, *DNMT3A*, *DNMT3B*, and the proliferation marker *MKI67* in the hCIMP and lCIMP ACC cell lines H295R (d) and MUC-1 (e). DMSO was used as the vehicle. Data represent the means from at least four independent experiments ± SEM (Wilcoxon test, *p < 0.05, **p < 0.01)

Expression levels of *DNMT1* and *DNMT3A* were, respectively, highly and moderately correlated with proliferation (Fig. 2a, b, Additional file 1: Fig. S2), corroborating the hypothesis that proliferation could also contribute to the increased expression of *DNMT* in hCIMP ACC tumors. As seen from the analysis of partial correlations, the association between cell proliferation and *DNMT* expression was statistically independent from the effect of copy number aberrations documented above (Fig. 2c, Additional file 3: Table S2), suggesting increased proliferation as a putatively independent cause of DNMT overexpression. Indeed, as mentioned earlier, hypermethylation has already been described or suggested to be a consequence of increased proliferation in prostate cancer [20] and acute myeloid leukemia [21]. The authors of the later paper suggested that this mechanism would be mediated by *DNMT3A*. In our model, we hypothesized that, as the DNMTs are regulated during the S phase [19], the hyperproliferation of ACC cells could drive an anarchic overexpression of these enzymes which would be found in excess in cells, inducing the methylation of portions of the genome that were not methylated in the parent cells.

To confirm that DNMT expression can be governed by increased cell proliferation, we treated hCIMP H295R and lCIMP MUC-1 ACC cell lines with an inhibitor of cell proliferation, AZD-5438, downregulating CDK1/2/9. The CIMP status of the ACC cell lines was established using the reduced representation bisulfite sequencing (RRBS) (see Methods). The AZD-5438 treatment negatively affected *DNMT3A* and *DNMT3B* expression in the hCIMP H295R cells; however, no decrease of *DNMT1* expression was observed (Fig. 2d, e). In contrast, no decrease of any *DNMT* expression following anti-proliferation treatment was observed in the lCIMP MUC-1 cells.

Mathematical modeling (see Methods) also corroborated our hypothesis that in ACC, DNA hypermethylation is driven by increased proliferation via augmented expression of *DNMT1* and *DNMT3A* (Fig. 2f). Indeed, partial correlations of the CIMP score and expression of the *DNMT* genes were significantly strong even when controlling for the cell proliferation status (corresponding partial correlation coefficients *R* and *p*-values: *R*_*DNMT1*_ = 0.29, *p* = 0.01;* R*_*DNMT3A*_ = 0.46,* p* = 3.08e−05) (Additional file 3: Table S2). Moreover, partial correlation of proliferation and CIMP score was not significant when controlling for the expression values of *DNMT1* and *DNMT3A* (*R* = 0.11, *p* = 0.35); of note, without conditioning for the DNMT expression values, the CIMP score was highly correlated with the cell proliferation scores (*R* = 0.51, *p* = 1.5e−06). The observation of such a low partial correlation excluded the hypothesis that cell proliferation can directly drive hypermethylation without the intermediation of over-expressed DNMTs and confirmed our thesis that DNTMs are direct drivers of CIMP.

### High and low CIMP tumors exhibit differences in proliferation and immune response gene expression signatures

Consistent with several earlier studies highlighting the aggressiveness of hCIMP ACC tumors [6, 7, 17], we observed strong associations of the CIMP score with metastasis (*R* = 0.29, *p* = 0.011), stemness score (*R* = 0.26, *p* = 0.02), and overall higher Weiss score (*R* = 0.30, *p* = 0.019) in the TCGA dataset. However, it was unclear whether these associations were direct and/or causal, or whether they were simply induced by the manifestation of CIMP in aggressive, highly proliferative cells (*R* = 0.51, *p* = 1.5e−06). Indeed, when controlling for the cell proliferation scores of the ACC tumors, these associations no longer held statistical significance (partial correlation of CIMP scores with metastasis *R* = 0.19, *p* = 0.10; stemness score *R* = 0.21, *p* = 0.07; Weiss score *R* = 0.07, *p* = 0.59).

To identify specific molecular processes and signaling pathways associated with CIMP in ACC, hCIMP tumors were compared to lCIMP tumors using Gene Set Enrichment Analysis (GSEA) [23]. Most of the gene sets enriched in hCIMP tumors were related to proliferation and/or cell survival, in line with our previous results (Additional file 1: Fig. S3 and Table 1). Notably, a large proportion of gene sets detected by GSEA as depleted in hCIMP compared to lCIMP tumors was associated with the immune system and immune response (50% of pathways with false-discovery rate (FDR) < 10% and negative normalized enrichment score), in addition to metabolism, oxidative phosphorylation, and protein secretion (Additional file 1: Fig. S3 and Table 1).

**Table 1:** Gene set enrichment analysis (GSEA) results for comparison of hCIMP and lCIMP samples

Name	Size	ES	NES	NOM *p*-val	FDR *q*-val
E2F_TARGETS	193	0.68	3.26	0	0
G2M_CHECKPOINT	194	0.67	3.18	0	0
MYC_TARGETS_V1	195	0.55	2.67	0	0
MYC_TARGETS_V2	58	0.54	2.1	0	0
MITOTIC_SPINDLE	199	0.42	2.03	0	0
CHOLESTEROL_HOMEOSTASIS	74	0.46	1.88	0	0.0004
DNA_REPAIR	149	0.41	1.84	0	0.0003
MTORC1_SIGNALING	198	0.35	1.69	0	0.003
PANCREAS_BETA_CELLS	40	0.45	1.63	0.008	0.005
UV_RESPONSE_UP	158	0.33	1.54	0.0038	0.011
UNFOLDED_PROTEIN_RESPONSE	113	0.3	1.34	0.039	0.072
SPERMATOGENESIS	135	0.29	1.31	0.044	0.083
PI3K_AKT_MTOR_SIGNALING	105	0.3	1.29	0.056	0.095
EPITHELIAL_MESENCHYMAL_TRANSITION	199	0.27	1.29	0.037	0.091
**INFLAMMATORY_RESPONSE**	**200**	**− 0.28**	**− 1.35**	**0.015**	**0.069**
**COAGULATION**	**138**	**− 0.29**	**− 1.36**	**0.027**	**0.065**
ESTROGEN_RESPONSE_EARLY	197	− 0.28	− 1.37	0.015	0.064
ADIPOGENESIS	200	− 0.29	− 1.39	0.011	0.062
KRAS_SIGNALING_UP	200	− 0.29	− 1.41	0.009	0.054
**IL6_JAK_STAT3_SIGNALING**	**87**	**− 0.33**	**− 1.43**	**0.019**	**0.052**
**COMPLEMENT**	**200**	**− 0.31**	**− 1.54**	**0**	**0.021**
PROTEIN_SECRETION	96	− 0.38	− 1.66	0.002	0.006
BILE_ACID_METABOLISM	112	− 0.37	− 1.66	0.002	0.008
FATTY_ACID_METABOLISM	156	− 0.37	− 1.73	0	0.004
OXIDATIVE_PHOSPHORYLATION	199	− 0.37	− 1.81	0	0.003
**INTERFERON_ALPHA_RESPONSE**	**97**	**− 0.46**	**− 2.03**	**0**	**0**
**ALLOGRAFT_REJECTION**	**200**	**− 0.46**	**− 2.21**	**0**	**0**
**INTERFERON_GAMMA_RESPONSE**	**200**	**− 0.45**	**− 2.21**	**0**	**0**

The MSigDB hallmark database was used and only the pathways with FDR < 10% are shown. Pathways with NES > 0 are enriched in hCIMP compared to lCIMP and pathways with NES < 0 are enriched in lCIMP compared to hCIMP. Pathways related to immunity are highlighted in bold. *ES* Enrichment Score, *NES* Normalized Enrichment Score

**Fig. 3:**
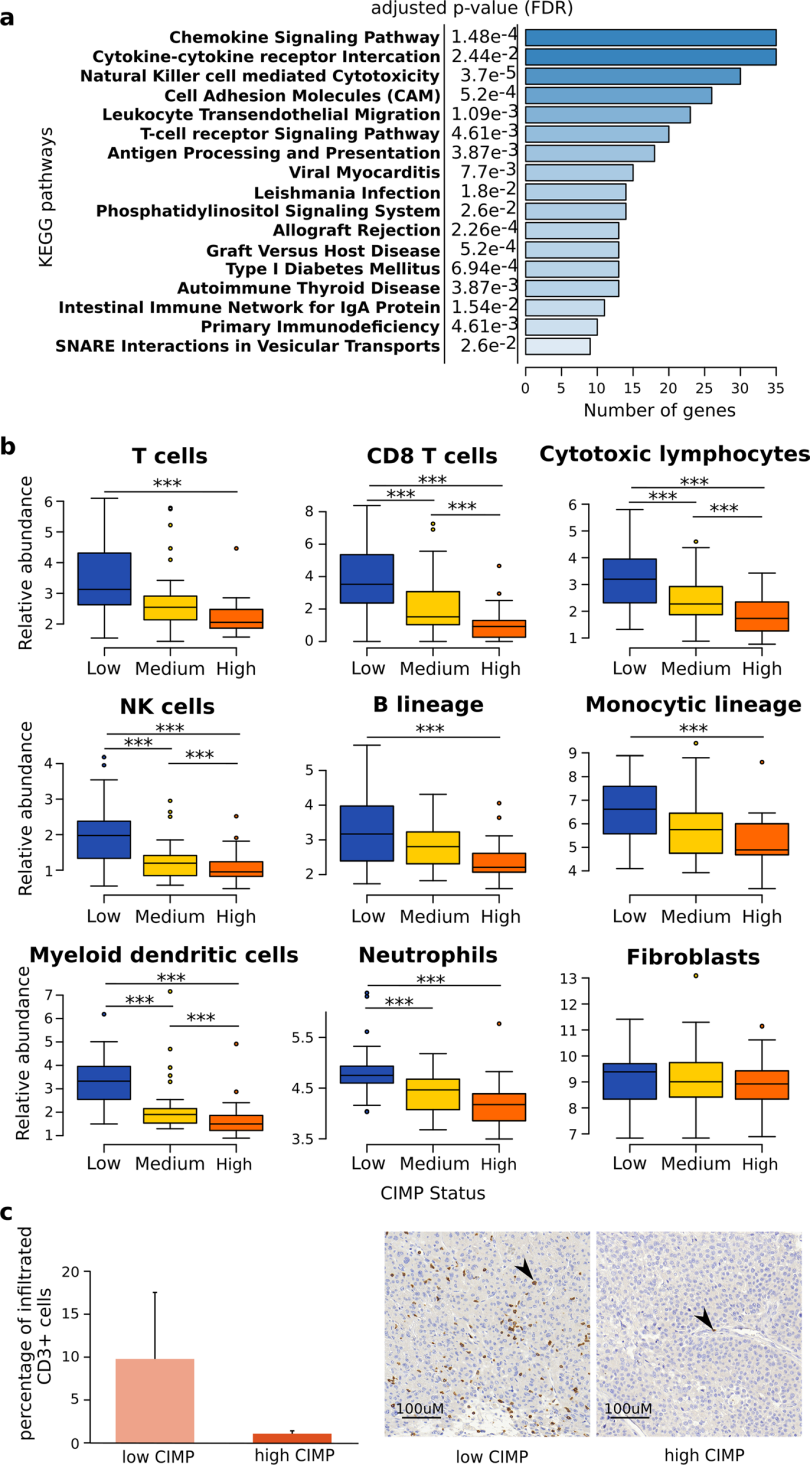
hCIMP ACC tumors are characterized by lower abundance of tumor-infiltrating immune cells. **a** Pathway enrichment analysis on genes repressed by DNA methylation in hCIMP tumors from TCGA dataset. The complete KEGG pathways database was used for the analysis and only the significantly enriched pathways are depicted on the figure. **b** Relative abundance of tumor-infiltrating immune and non-immune stromal cell populations computed using MCP-counter [19] in lCIMP (*n* = 32), iCIMP (*n* = 26), and hCIMP (*n* = 21) samples from TCGA dataset. Comparison of the relative abundances of each population using the Kruskal–Wallis test followed by Dunn's test with Benjamini–Hochberg corrections. ***p < 0.001. **c** Left: quantification of the percentage of tumor-infiltrating CD3 + T cells. Data represent the means from at least six independent experiments ± SEM. *p* value of the Mann–Whitney *U* Test: *p* = 0.12. Right: representative screenshot of a highly infiltrated lCIMP sample and a lowly infiltrated hCIMP sample

To check whether indeed increased DNA methylation might be responsible for the immune escape, we screened for molecular processes downstream of CIMP. To identify genes repressed by DNA methylation in hCIMP, we selected genes whose expression levels significantly correlated with the CIMP status and whose promoters or gene bodies contained differentially methylated cytosines (See Methods). A total of 1302 genes were found to be potentially repressed by methylation in CIMP. A pathway enrichment analysis was performed on all KEGG pathways and showed that almost all pathways negatively influenced by DNA methylation-dependent repression of transcription were related to immune response or communication with the immune system (Fig. 3a). We therefore conclude that CIMP can directly cause inhibition of immunity-related genes.

### hCIMP ACC tumors are characterized by low infiltration of immune cells

Given the differences uncovered in immune response gene expression between lCIMP and hCIMP tumors, we hypothesized that hCIMP tumors could be less infiltrated by immune cells than lCIMP tumors. To test this hypothesis, abundance of tumor-infiltrating immune cells in ACC tumors was estimated in silico using MCPcounter software [24] for both TCGA and ENSAT cohorts. Abundance of the eight assessed immune cell types was significantly decreased in hCIMP compared to that in lCIMP tumors whereas fibroblast abundance was comparable among the tumor groups (Fig. 3b and Additional file 1: Fig. S4).

To validate the decrease in tumor infiltrating immune cells in ACC, we performed immunohistochemistry experiments using CD3 as a marker of T cells on 6 hCIMP and 6 lCIMP tumors (Additional file 4: Table S3). We found that the lCIMP group of tumors contained a few highly infiltrated samples, while all hCIMP were all poorly infiltrated (Fig. 3c). In sum, we concluded that DNA methylation of immune response genes can affect the composition of tumor-infiltrating immune cells in the hCIMP ACCs.

### Treatment of the hCIMP ACC cell line with a DNA demethylating agent re-activates genes related to the immune system

**Fig. 4:**
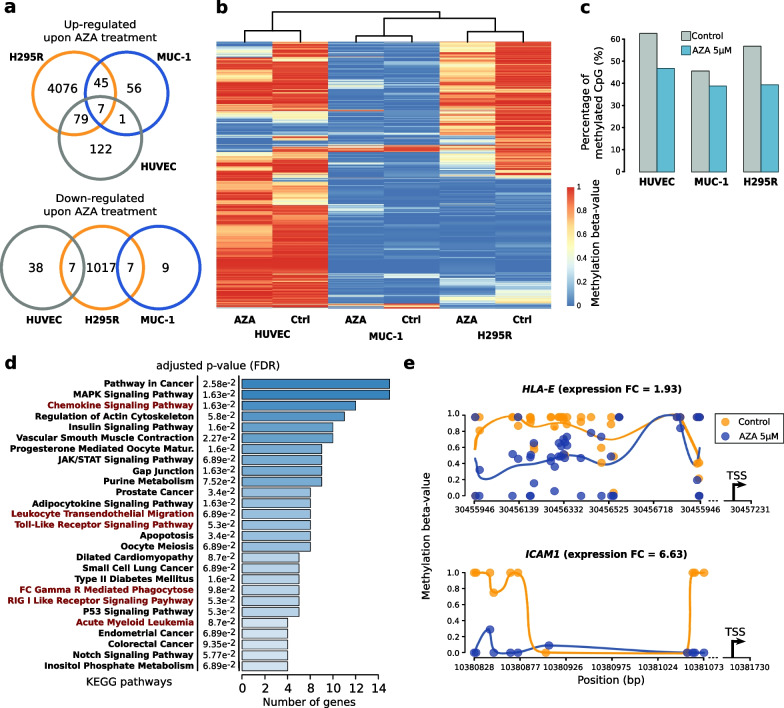
Demethylating agent 5-azacytidine (AZA) can partially revert DNA hypermethylation of CpG islands in the hCIMP ACC cell line H295R restoring activity of immune response genes. **a** Overlap between genes significantly up- or down-regulated upon AZA treatment (FDR < 0.05) in H295R (5242 genes), MUC-1 (125 genes), and HUVEC (254 genes) cell lines. **b** Unsupervised hierarchical clustering of 3 cell lines (H295R, MUC-1 and HUVEC) treated or not with AZA 5 μM was based on their promoter CpG islands methylation profile. The clustering was obtained using the CpG with the most variable methylation level (s.d. > 0.4). **c** Global percentage of methylated CpG among the CpG covered by RRBS reads. AZA induces a decrease in CpG methylation in all three cell lines, but this effect is less pronounced in the MUC-1 cell line that has the lowest basal level of DNA methylation. **d** Pathway enrichment analysis on genes induced by AZA in H295R through promoter demethylation. Pathways related to the immune response are highlighted in red. **e** Examples of differentially methylated region (DMRs) upon AZA treatment in H295R located in promoters of two immune response genes

Our results led to the hypothesis that DNA methylation in hCIMP ACC could reduce antitumor immunity. Thus, reversing this effect of DNA methylation might be beneficial for patients by reintroducing antitumor immunity and favoring the action of immunotherapy. To test this hypothesis, we inhibited DNA methylation in ACC cells by treating the hCIMP H295R and lCIMP MUC-1 cell lines with the demethylating agent 5-azacytidine (AZA) (5 µM) and assessed the resulting expression and methylation profiles using RNA-Seq and RRBS, respectively. The primary human umbilical vein endothelial cell line (HUVEC) was used as a non-ACC control. AZA treatment resulted in decreased CpG methylation in all three cell lines associated with mainly positive changes in gene expression (Fig. 4a-c and Additional file 5: Table S4). However, effects of treatment were less marked in MUC-1 cells in accordance with the global DNA hypomethylation of this cell line (Fig. 4b, c). Of note, the hCIMP H295R cell line was significantly more sensitive to the AZA treatment than the lCIMP MUC-1 and control HUVEC cell lines (Additional file 6: Fig. S5).

For each cell line, the ability of AZA to reverse the methylation-dependent repression of gene expression was assayed by extracting the genes for which AZA was able to decrease promoter methylation while inducing an increase in expression (see Methods). A total of 560, 13, and 48 genes were identified for H295R, MUC-1, and HUVEC cell lines, respectively (Additional file 5–8: Tables S4–S7). As previously observed, AZA dependent decrease in CpG methylation had a larger effect in H295R cells than in the hypomethylated MUC-1 line. Notably, treatments of the non-ACC control HUVEC cells with AZA resulted in the re-expression of approximately fourfold fewer genes than in H295R cells even though the former cell line exhibited higher initial DNA methylation levels.

Interestingly, the 560 genes demethylated and reactivated by AZA in the hCIMP model H295R included several genes involved in the interaction of cancer cells with the tumor microenvironment such as *HLA-E, ICAM1* and *RAPGEF3* (Additional file 6: Table S5). Pathway enrichment analysis further revealed that 27 pathways were enriched in H295R cells upon DNA demethylation (FDR < 0.1) including several pathways related to immune response and cell death (Fig. 4d, e). In comparison, only 0 and 2 pathways were found to be enriched in the MUC-1 and HUVEC gene sets, respectively, owing to the low number of differentially expressed genes in these sets (Additional file 9: Table S8).

As these observations suggested that AZA is able, in the hCIMP cellular model, to induce gene re-expression through the direct promoter demethylation of genes involved in similar immune pathways as the genes repressed by methylation in hCIMP ACCs, we evaluated the genes that were found in both analyses in further detail. Overall, 47, 6, and 11 genes were identified both as genes induced by AZA through promoter demethylation and those repressed in hCIMP tumors through promoter methylation, respectively, in H295R, MUC-1, and HUVEC cell lines. A full listing and brief description of the functions of these genes is provided in Additional file 10: Table S9. Notably, the list for H295R cells contained not only several genes related to immune system but also some known or putative tumor suppressor genes such as *ARHGAP10*, *HCG11*, *LOXL4*, and *LRIG1*.

Using the same approach, we determined the genes repressed by promoter hypermethylation in H295R compared to MUC-1 cells in the absence of treatment. Notably, several pathways enriched for this gene set were also related to the immune system-associated genes, confirming that these two cell lines recapitulate at least partially the molecular phenotypes observed in hCIMP and lCIMP tumors (Additional file 1: Fig. S6 and Additional file 11: Table S10). In addition, this observation also supported the results of our analysis of public datasets demonstrating that in ACC, DNA methylation-dependent gene repression targets the immune system in hCIMP compared to lCIMP tumors.

## Discussion

In this study, we investigated the role of augmented CpG island DNA methylation (CIMP) and its driving mechanisms in ACC. It has been known that unlike other cancer types with CIMP [25, 26], ACCs do not harbor mutations in DNA methylation-related genes [6, 7]. Our re-analysis of ENSAT exome sequencing data using a more permissive threshold of mutation confidence (to identify sub-clonal mutations) has not been able to discover any such mutations either (data not shown). Here, we proposed that augmented CpG island DNA methylation in this cancer type is associated with the increased expression of *DNMT1* and *DNMT3A*, as previously reported in some other cancer types such as gastric cancer and gliomas [27, 28]. We revealed that increased *DNMT1* and *DNMT3A* expression was associated with gains of copy number of these genes in hCIMP compared to lCIMP tumors along with higher cell proliferation in hCIMP tumors.

Analysis of TCGA and ENSAT datasets also revealed a link between DNA methylation and tumor immunity in ACC. Pathway analysis showed that activities of many pathways linked to immunity were decreased in hCIMP as compared to lCIMP, in particular, those linked to inflammation (e.g., inflammatory response, JAK STAT signaling pathway, and interferon alpha/gamma) (Table 1). Indeed, it has been well described that activation of the JAK STAT pathway leads to the activation of the expression of the interferons [29]. Interferons then make the tumor an easier target for the immune system by activating NK cells and cytotoxic lymphocytes and enhancing the presentation of tumor-associated antigens [30, 31]. Besides the pathways linked to immune system, some pathways that had been suspected to be associated with DNA hypermethylation or DNMT activity, such as DNA repair, proliferation/cell cycle pathways and AKT pathways [32], were also enriched in hCIMP as compared to lCIMP (Table 1). However, in ACC, they do not seem to be directly regulated by DNA methylation (Fig. 3a).

Indeed, strikingly, most of the pathways enriched among genes directly silenced by DNA methylation were also associated with immunity (Fig. 3a). This list included “antigen presentation and processing” pathway on account of methylation-related silencing of many *HLA* genes, a mechanism of immune escape shared by several cancer types [33, 34]. Several pathways involved in immune cells activation and recruitment were also detected by GSEA as directly affected by DNA methylation in hCIMP (e.g., chemokine signaling, cytokine-cytokine receptor interaction, and natural killer cell mediated cytotoxicity). These results were further confirmed by a deconvolution of immune cell abundance analysis using expression signatures. And although it could be interesting in future to perform single cell RNA-seq to get a more accurate estimation of the tumor infiltrating immune cells, our immunohistochemistry experiments have also confirmed the low immune status of hCIMP samples. Tumor-infiltrating immune cells can limit tumor progression, and deficiency of immune cells has been often observed in aggressive and/or metastatic tumors [35, 36]. Interestingly, a link between DNA methylation and tumor infiltration by immune cells has already been described in several other cancer types, such as gliomas, colorectal cancers and ependymomas [14, 37, 38].

Our treatment of ACC cells with AZA resulted in a partial reversion of the methylation-dependent silencing of immune gene expression in the hCIMP H295R cells. In particular, *HLA-E*, which was repressed by methylation in hCIMP tumors, was significantly induced by AZA. *HLA-E* was found to contain a differentially methylated region (DMR) hypomethylated under AZA treatment. Our observations fit into the context of several other cancer studies. For example, it has been shown that the expression of *HLA* genes is lost in several cancer types and this loss contributes to the aggressiveness and the immune evasion of these tumors [13, 39]. Moreover, loss of *HLA* gene expression has been associated with promoter hypermethylation and has been shown in some cases to be reversible by AZA or decitabine treatment [39, 40].

Overall, many genes and pathways involved in immune cell mobility and chemokine/cytokine production were re-expressed after treatment upon decreased in promoter methylation (Additional file 6 and 9: Tables S5 and S8). Thus, in our suggested model, methylation-dependent silencing of immune genes, including previously mentioned genes involved activation and recruitment of immune cells, would impair anti-tumor immunity leading to the observed reduction in tumor-infiltrating immune cells and this would contribute to the lower prognosis in high CIMP ACC patients. Even though further study needs to be conducted, our result on cell lines strongly suggests that treatment with the demethylating agent azacytidine could at least partially reverse this mechanism as it has been shown to induce the re-expression of genes involved in immune pathways. For example, are found among these re-expressed genes, *HLA-E* mentioned in the previous paragraph but also *RAPGEF3*, whose expression has been correlated with neutrophil infiltration in uveal melanoma [41], *ICAM1*, whose down-regulation is a known mechanism of immune escape during tumorigenesis [42], and *RIPK1*, whose methylation dependent silencing increases tumorigenic properties and migration abilities of cancer cells in head and neck squamous cell carcinoma [43]. Of note, gene expression changes following the AZA treatment were also observed in the healthy control cell line HUVEC and in the lCIMP ACC cell line MUC-1, albeit at a significantly lower scale compared to the hCIMP ACC cell line H295R (254, 125 and 5242 significantly deregulated genes, respectively) (Fig. 4a). We also observed a much higher sensitivity of the hCIMP H295R cell line to the AZA treatment than the lCIMP MUC-1 and the non-cancerous HUVEC cell lines. However, given the fact that MUC-1 is generally much more resistant to most types of treatment than H295R, one should not extrapolate these findings on AZA sensitivity in cell lines to ACC tumors broadly [44–46].

Several clinical trials have been carried out to assess the effectiveness of immunotherapies in treating ACC [47]. However, none of the trials combined immunotherapy with DNA demethylating agents, and all tested agents have shown modest clinical activity so far. Based on our findings, it would be interesting in future to design a clinical trial combining the two mechanisms to treat the aggressive hCIMP ACC tumors.

Strikingly, the expression of *CD274* (encoding the major immune checkpoint protein PD-L1) was also enhanced under AZA treatment in ACC even though no DMRs were identified (fold change 1.77, FDR 0.0389); the latter may be due to the low number of RRBS reads mapping to the *CD274* promoter. Several studies have suggested that DNMT inhibitors, such as AZA, could be used to enhance the clinical success of immunotherapy, e.g., the efficacy of PD-L1 inhibitors [48]. Notably, re-expression of *CD274* following AZA treatment has been shown to sensitize patients with non-small cell lung cancer to immune checkpoint inhibitors [49, 50]. The results of our study thus strongly support the use of DNMT inhibitors in combination with immunotherapy in the context of ACC, especially in the case of patients with hCIMP. Indeed, this combination of treatments could help overcome the limited results obtained by different studies and clinical trials that have evaluated the effect of immunotherapy in ACC [51–53].

In addition to DNA methylation, posttranslational histone modifications are known to have a critical role in chromatin stability in cancer [54]. However, our recent study of histone modification landscape in ACC did not show any strong association between the locally present histone modifications (H3K27ac, H3K27me3, and H3K4me3) and DNA methylation [55]. Our analysis of super-enhancer landscape did not identify any super-enhancer elements in *DNMT* genes in ACC, which could explain *DNMT* over-expression in CIMP cases. Neither could we link the CIMP pattern and global variation in H3K27ac or H3K27me3 in ACC tumors. The only significant association was observed between the ACC CIMP and broad H3K4me3 regions, suggesting avenues for further studies.

In addition to the increased expression of *DNMT1* and *DNMT3A* in the hCIMP tumors, we reported significantly higher expression of the *TET1* gene, which codes for a protein involved in DNA demethylation. Although we do not have a clear explanation for this phenomenon, we hypothesize an existence of a controlling feedback loop between the expression of genes linked to DNA methylation and demethylation. This hypothesis is in line with the strong correlation values observed in the pan-cancer TCGA dataset (*TET1* vs. *DNMT1*, *R* = 0.228, *p* = 4.76e−55; *TET1* vs. *DNMT3A*, *R* = 0.443, *p* = 5.14e−218). We, however, argue that increased *TET1* expression in hCIMP cases is unlikely to compensate for the increased expression of DNMTs given expression levels of these genes (mean expression in hCIMP tumors of TCGA (RPKM values): *DNMT1* = 1664; *DNMT3A* = 1345; *TET1* = 93).

A limitation of our study is that we made use of only two cell lines as ACC cellular models: H295R and MUC1. However, only a very small number of ACC cell lines is currently available owing to the difficulties of cell line establishment for this cancer. Nevertheless, the chosen cell lines, H295R and MUC1, well recapitulate lCIMP and hCIMP. Another limitation of our study is linked to the scarcity of the tumor material we could obtain to quantify immune cell infiltration in ACC. Indeed, we could only evaluate proportions of infiltrating T cells (expressing the CD3 marker) in 12 ACC patients without distinguishing T cell subtypes. In future, it will be valuable to experimentally assess the immune infiltration levels for a larger number of ACC tumors using such markers as CD4, CD8, FOXP3 for subtypes of T cells and other markers for different populations of immune cells.

**Fig. 5:**
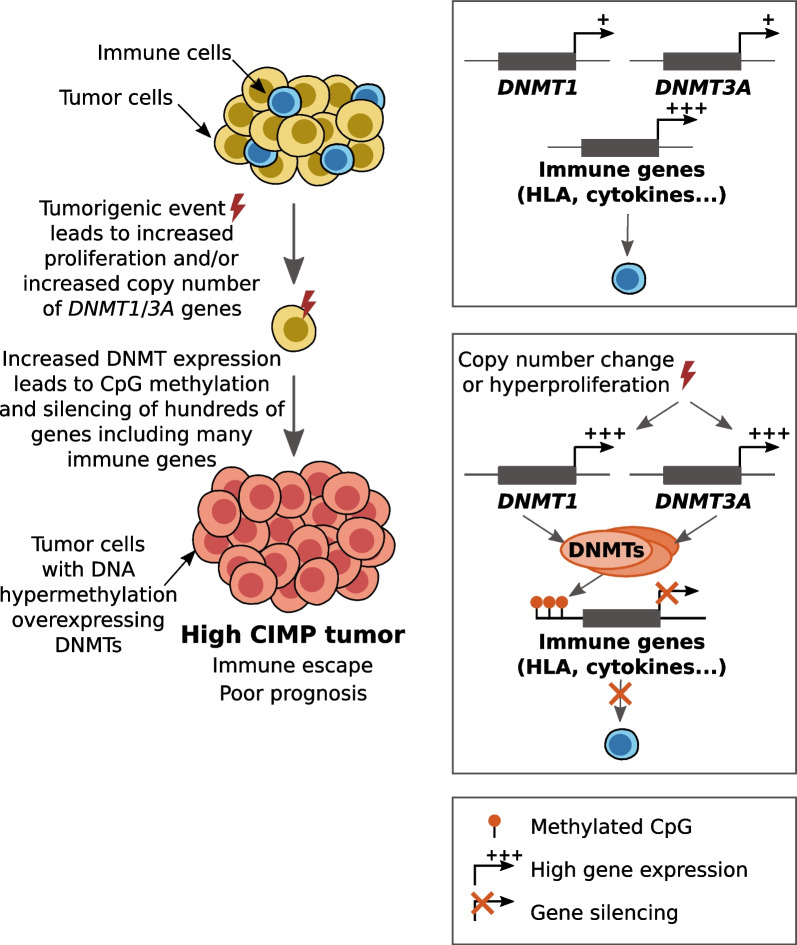
Suggested model of the establishment of the high CIMP in ACC. Augmented expression of DNA methyltransferases DNMT1 and DNMT3A, driven by genomic aberrations and hyperproliferation, results in an increase of promoter DNA methylation of hundreds of genes; consequently, methylation-dependent silencing of genes related to immune response leads to lower infiltration of the tumor by immune cells

To the best of our knowledge, this is the first study that attempts to decipher the causes and consequences of CIMP in ACC. Taken together, our results shed light on mechanisms involved in establishment of this phenotype; i.e., increased expression of *DNMT1* and *DNMT3A* as a consequence of augmented gene copy number and hyperproliferation. Furthermore, we showed that CIMP has a direct effect on the aggressiveness of tumors through favoring the tumor immune escape (Fig. 5). We also showed that this effect could be at least partially reversed by treatment with the demethylating agent AZA. Together, our results strongly suggest that the co-treatment of patients with hCIMP ACC with DNA demethylating agents and immunotherapy drugs may positively affect patient survival.

## Methods

### Public data analysis

We used two independent cohorts of ACC tumors: 40 ACC samples from ENSAT characterized by transcriptomic microarrays (Gene Expression Omnibus (GEO) dataset GSE49278) and Illumina Infinium HumanMethylation27k assays (GEO dataset GSE49277); 79 ACC samples from TCGA (https://portal.gdc.cancer.gov/) cohort including mRNA sequencing data and Illumina Infinium HumanMethylation450k assays. In both cohorts, the authors identified subgroups of patients with low, intermediate, and high CIMP (lCIMP, iCIMP and hCIMP, respectively). Patients from the two cohorts assigned to similar DNA methylation groups shared other molecular characteristics such as patterns of copy number alterations, point mutations and gene expression profiles. Clinical and demographics data of the tumors/patients included in this study are available in Additional file 12 and 13: Tables S11 and S12 and more exhaustive information can be found in the corresponding papers [6, 7].

GSEA [23] was performed on expression data using the signal-to-noise ratio ranking method and the hallmark gene sets from the Molecular Signatures Database (MsigDB v6.2) [56]. Pathway enrichment analysis using unranked gene lists was performed using ACSNmineR (v0.16.8.25) [57].

### Computation of the “CIMP score”

For subsequent analysis, we obtained a quantitative value of “CIMPness,” the CIMP score, based on DNA methylation assays from TCGA. First, CpG probes located in promoters of genes were extracted (“TSS200” in the Illumina Infinium HumanMethylation annotation file). Probes that were found to differ significantly between lCIMP, iCIMP, and hCIMP (as defined in [6]) with a delta β-value > 0.2 between lCIMP and iCIMP samples and between iCIMP and hCIMP were selected as CIMP signature probes. The score was then calculated as the sum of the β-values of these probes.

### Estimation of the abundance of immune cell populations

For TCGA dataset, RSEM normalized read counts were first log scaled (log2(x + 1)) and then processed to estimate the population of ten immune cell populations using the R package MCP-counter [24]. These ten populations included T cells, CD8 + T cells, cytotoxic lymphocytes, NK cells, B lineage, monocytic lineage, myeloid dendritic cells, neutrophils, endothelial cells, and fibroblasts. The relative abundance of each population was compared between CIMP groups. Statistical significance values of the observed differences in abundance were evaluated using the Kruskal–Wallis test, followed by the Dunn tests with FDR corrections.

### Stemness score calculation

We calculated the stemness score as a mean gene expression value for the previously reported stemness-related marker genes: *OCT4*/*POU5F1*, *SOX2*, *KLF4*, *C-MYC*, *NANOG*, *SALL4* [58].

### Modeling the link between DNA methylation, expression of *DNMT1* and *DNMT3A* and cell proliferation

We observed strong positive correlation between the expression of *DNMT1*, *DNMT3A*, the CIMP status and cell proliferation (Fig. 2a-b). Analysis of partial correlations was employed to decipher connections between these covariates. In this analysis, the CIMP score was corrected for tumor purity values; however, this qualitatively did not change the results.

Analysis of partial correlations can identify associations induced by the third factor. We applied this approach to prove the consistency of the model that in ACC cell proliferation causes the increase in expression of DNMTs, which in turn results in augmented DNA methylation (i.e., increased CIMP score). First, significant partial correlation of the CIMP status with DNTM expression when controlling for cell proliferation excluded the possibility that cell proliferation is a common cause for both increased DNMT expression and CIMP (Additional file 3: Table S2). Therefore, we assumed that DNMTs cause CIMP independently from cell proliferation.

Second, we tested the model that cell proliferation drives CIMP through augmenting expression of DNTMs by calculating partial correlation between cell proliferation and CIMP score when controlling for the *DNMT1* and *DNMT3A* expression. Observed partial correlation between cell proliferation and CIMP score was small and not significant (Additional file 3: Table S2), which showed the consistency of the proposed model. We concluded that the TCGA ACC data confirmed the model that increased cell proliferation causes augmented expression of DNMTs, which in turn cause an increase in DNA methylation at CpG islands (CIMP).

### Tumor samples

Tumor samples for immunohistochemistry were collected between 2008 and 2015 in the Cochin hospital (Paris, France), snap-frozen, and stored as previously reported [7]. Signed informed consent was obtained from all patients and the study was approved by the local institutional review board (Comité de protection des personnes Ile de France 1, application #13311).

### Cell lines and treatments

Human H295R cells were cultured in Dulbecco’s modified Eagle medium (DMEM)/F12 with 15 mM HEPES and sodium bicarbonate, without L-glutamine (Sigma-Aldrich, St. Louis, MO, USA), supplemented with 2% NU-serum (Corning, Chorges, France), 1% insulin, transferrin, and selenium (100 U/mL; Gibco, Gaithersburg, MD, USA), penicillin/streptomycin (100 U/mL; Gibco), and 1 mL plasmocin (2.5 mg/mL, Invivogen, San Diego, CA, USA). The cells were grown in humidified 5% CO_2_ at 37 °C. Human MUC-1 [44] cells were cultured in Advanced DMEM/F12 Medium (GIBCO) supplemented with 10% fetal bovine serum and 1% penicillin/streptomycin and grown in humidified 5% CO_2_ at 37 °C. Human HUVECs were cultured in DMEM with glutamax, 4.5 g D-glucose, and pyruvate (Gibco) supplemented with 10% fetal bovine serum and penicillin/streptomycin (100 U/mL; Gibco) in a humidified incubator containing 5% CO_2_ at 37 °C. H295R and HUVEC cell lines were purchased from the ATCC. MUC-1 cells were obtained from Constanze Hantel (UniversitätsSpital Zürich, Zurich, Switzerland). Whole genome sequencing revealed that H295R exhibits some driver genes mutations often seen in ACCs (*CTNNB1*, *ZNRF3*, *RB1* and *TERT*) as well as some characteristic copy number variations including amplifications in the 9q, 19q and 12p regions and loss in the 22q region. The MUC-1 cell line did not carry any of the common mutations in ACC but exhibited a copy number profile typically described as chromosomal with gains and losses of large chromosomal regions, including full arms or chromosomes.

The proliferation inhibitor AZD-5438 (Cliniscience, Nanterre, France) and the demethylating agent AZA were used at different doses and times depending on the experiments. The solvent (DMSO) was used as a control.

### Characterization of the methylation status of cell lines

The CpG methylation levels of all cell lines were assayed by RRBS (see below). Methylation levels were found to be relatively similar between H295R cells and HUVECs, whereas MUC-1 cells [44] were characterized by global DNA hypomethylation (Fig. 4b, c). In particular, CpGs within promoter CpG Islands were strongly hypomethylated in MUC-1 cells as compared to H295R cells, leading us to consider MUC-1 cells as an lCIMP model and H295R cells as hCIMPs (Fig. 4b, c).

### RNA extraction

Total RNAs extractions were performed using TRIzol® Reagent (Invitrogen, Carlsbad, CA, USA) according to manufacturer's protocol. Extracted RNAs were then treated with DNAse (TURBO DNA-free™ kit, Invivogen) following provider’s instructions. MRNA were then used for RT-qPCR analysis or RNA-Seq.

### Quantitative reverse transcription polymerase chain reaction (RT-qPCR)

Reverse transcription was carried out from 2.5 µg of total RNA, first mixed with dNTP (2 µL, 10 mM, Invitrogen) and random primers (1 µL, 50 µM, Invitrogen) and incubated at 65 °C for 5 min. After hybridization, samples were held on ice prior to the addition of 1 µL DEPC-treated H_2_O (Ambion, Austin, TX, USA), 1 µL 0.1 M dithiothreitol (Invitrogen), and 4 µL 5X buffer (Invitrogen). RNAse inhibitors (1 µL), RNAse out (40 U/µL, Invitrogen), and reverse transcriptase (1 µL, SuperScript IV, Invitrogen) were added and the reaction was incubated at 25 °C for 10 min, followed by an incubation at 42 °C for 40 min. qPCR experiments were performed using the SensiFAST™ SYBR® No-ROX Kit (Bioline) with a LightCycler480 apparatus (Roche, Roswell, GA, USA).

### Choice of the normalizing genes for RT-qPCR analysis

Normfinder [59] was used to find the normalizing genes *ACTG1* and *GAPDH*. The sequences of primers used are available upon request. Relative gene expression was determined by normalizing raw data for the control gene and the control sample using the DDCt method.

### RNA Sequencing (RNA-seq)

For experiments on cell lines, approximately 3 × 10^5^ cells were used, treatments were performed in triplicate, and RNA-Seq experiments were performed at least in duplicate. Total RNA was extracted and treated with DNAse as described in the RT-qPCR section. Quality controls were performed using a Bioanalyzer (Agilent, Santa Clara, CA, USA) before and after library preparation and only RNA samples with RNA Integrity Number > 6 were used for sequencing.

For sequencing library preparation, 1 µg of high-quality total RNA samples was used except for the AZA treatment experiments, for which 1% ERCC spike-in RNA (Life Technologies, Carlsbad, CA, USA) was added to 3 µg of high-quality total RNA samples. Notably, AZA treatment was expected to result in a global increase in gene expression, thus impairing usage of library size normalization. Sequencing libraries were prepared using the TruSeq Stranded mRNA kit (Illumina) according to the manufacturer’s instructions. Briefly, after purification of poly-A-containing mRNA molecules, mRNAs were fragmented and reverse-transcribed using random primers. dTTP was replaced by dUTP during the second strand synthesis to achieve strand specificity. Addition of a single A base to the cDNA (dA tailing) was followed by ligation of Illumina adapters. Libraries were quantified using the Qubit fluorometer (Life Technologies) and library profiles were assessed using the DNA high Sensitivity LabChip kit on an Agilent Bioanalyzer 2100. Libraries were sequenced on an Illumina Nextseq 500 instrument using 75 base-length-read chemistry in a paired-end mode. After sequencing, primary analysis based on AOZAN software [60] was applied to demultiplex and control the quality of the raw data.

Mapping of the reads to the human reference genome hg19/GRCh37 was performed using STAR v2.5.3a [61] with the following parameters: twopassMode, “Basic”; chimSegmentMin, 12; chimJunctionOverhangMin, 12; alignSJDBoverhangMin, 10; alignMatesGapMax, 100,000,000; alignIntronMax, 200,000; alignSJstitchMismatchNmax, 5 -1 5 5. FeatureCount [62] was used to retrieve the gene-level raw read counts from aligned reads and DESeq2 [63] was used to normalize the data and find differentially expressed genes. For spike-in data, raw read counts were normalized using the function RUVs from the R package “RUVSeq” (“remove unwanted variation”, v1.16.1) [64] and then processed with DESeq2 v1.22.2 without size factor normalization to identify differentially expressed genes.

### RRBS experiments

Genomic DNA was extracted using the QIAamp® Fast DNA Tissue Kit (Qiagen) according to the manufacturer’s protocol. RRBS was performed by Integragen SA (Evry, France) using the Diagenode Premium RRBS kit. In brief, 100 ng of qualified genomic DNA was digested with MspI. After end-repair, A-tailing, and ligation to methylated and indexed adapters, the size-selected library fragments were subjected to bisulfite conversion, amplified by PCR, and sequenced on an Illumina NovaSeq sequencer as Paired End 100 bp reads. Image analysis and base calling were performed using Illumina Real Time Analysis (3.4.4) with default parameters. Base calling was performed using the Real-Time Analysis software sequence pipeline (3.4.4) with default parameters. RRBS data were mapped to the Human genome (hg19) using BS-Seeker2 v2.1.8 [65]. The following parameters were used: -r (map reads to the Reduced Representation genome), -c C-CGG (MspI: sites of restriction enzyme and specifying lengths of fragments ranging [40, 400 bp]. One mismatch was allowed in the adaptor sequence. Allowing local/gapped alignment with Bowtie2 [66] increased the mappability.

The BS-Seeker2 module bs_seeker2-call_methylation.py was used to call methylation levels from the mapping results with these parameters: –rm-SX (removed reads that would be considered as not fully converted by bisulfite) and –rm-overlap (removed one mate if two mates overlapped). The methylation callings were saved in the ATCGmap/CGmap format. Cgmaptool v0.1.2 [67] was used to call DMRs.

Genes for which AZA was able to decrease promoter methylation while inducing an increase in expression were retrieved. These were defined as genes (1) having at least one DMR within their promoters (defined as the region including 1000 bp before the TSS and 200 bp after the TSS) with significant *p* value (< 0.05) and a delta between control and AZA > 0.2 and (2) being significantly upregulated by AZA with *p* value < 0.05 and fold change > 2.

### Immunohistochemistry

Immunohistochemistry for CD3 was performed on paraffin-embedded tissues (6 hCIMP and 6 lCIMP tumors) in paraffin using a Leica Bond III automat. Samples were deparaffinized and unmasked at pH 6 and then immunostained using the Bond Polymere Refine kit (Leica, Wetzlar, Germany). The antibody used was anti-CD3 ab16669 (rabbit polyclonal, Abcam). Slides were scanned with a Lamina slide scanner (PerkinElmer, Waltham, MA, USA) and CaseViewer (3DHISTECH) was used to obtain images. Number of stained cells and total number of nuclei in at least 10 different fields per sample were counted using the GNU Image Manipulation Program (GIMP; https://www.gimp.org/).

### Statistical analysis

All statistical analyses were performed using R (22). Pearson and Spearman correlation tests, in addition to Wilcoxon and Kruskal–Wallis tests, do not need the data to follow normal distribution nor require homoscedasticity. When not stated otherwise, *p* values < 0.05 were considered significant.

## Supplementary Information

**Additional file 1: Fig. S1.** related to content in Fig. 1: DNMT1 and DNMT3A expression levels are upregulated in high CIMP ACC and are associated with poor clinical outcome. (a) Expression levels of DNMT1, DNMT3A, DNMT3B, TET1, TET2 and TET3 in the ENSAT patients exhibiting lCIMP (*n* = 20), iCIMP (*n* = 14) and hCIMP (*n* = 6) (two-sided *t* test, ***p < 0.005). (b) Kaplan–Meier estimates of overall survival for ACC patients, as a function of DNMT1 or DNMT3A. **Fig. S2** related to content of Fig. 2: *DNMT1* and *DNMT3A* expression is associated with high proliferation. Correlation between *DNMT1* and *DNMT3A* expression and cell proliferation in the ENSAT patients. hCIMP (*n* = 6) samples are represented in red, iCIMP (*n* = 14) in orange and lCIMP (*n* = 20) in blue. Patients for whom the CIMP status was not defined are represented in gray. **Fig. S3** related to content of Table 1: Gene Set Enrichment Analysis on TCGA datasets comparing hCIMP and lCIMP samples. (a) Representative example of gene sets enriched in hCIMP. (b) Representative example of gene sets enriched in lCIMP. **Fig. S4**, related to content of Fig. 3: CIMP in ACC is characterized by lower abundance of tumor-infiltrating immune cells Relative abundance of tumor-infiltrating immune and non-immune stromal cell populations, computed using MCP-counter, in lCIMP (*n* = 20), iCIMP (*n* = 14) and hCIMP (*n* = 6) samples from the ENSAT dataset. Comparison of relative abundances of each population using Kruskal–Wallis test followed by Dunn's test with Benjamini–Hochberg corrections. **Fig. S5** related to content of Fig. 4: Impact of demethylating agent on cell proliferation in ACC cell lines. Effect of a 6-day treatment with the DNA methylation inhibitor 5-azacytidine (AZA) at 1, 5, or 10 µM in the H295R (*n* = 4), MUC1 (*n* = 5) cell lines and the non-tumor HUVEC cell line (*n* = 5). Cell growth was estimated by neutral red proliferation assay. *p* values of the Kruskal–Wallis test and Dunn’s test for stochastic dominance are reported: *p value < 0.05; **p value < 0.01; ***p value < 0.005. **Fig. S6** related to content of Fig. 4: Gene pathways differentially active in H295R compared to MUC-1 cells due to DNA hypermethylation. Pathway enrichment analysis on genes that exhibit a significantly hypermethylated DMR in their promoter and significantly lower expression in H295R than in MUC-1 cells. Pathways related to the immune response are highlighted in red.

**Additional file 2: Table S1** related to content in Fig. 1: Genes coding for factors known to be involved in DNA methylation or demethylation.

**Additional file 3: Table S2** related to content in Methods: Partial correlation analysis for modeling interactions between increased cell proliferation, expression of DNMTs and CIMP status (TCGA data set). DNMT expression is measured in log2(RPKM + 1).

**Additional file 4: Table S3** related to content in Fig. 3: Characteristics of the ACC cohort used to perform immunohistochemistry experiments using CD3 as a marker of T cells including 6 hCIMP and 6 lCIMP tumors.

**Additional file 5: Table S4** related to content in Fig. 4: Differential expression analysis using DESeq2 as a result of sample treatment with AZA (5-azacytidine).

**Additional file 6: Table S5** related to content in Fig. 4: Differential expression and methylation analysis as a result of sample treatment with AZA (5-azacytidine) in H295R.

**Additional file 7: Table S6** related to content in Fig. 4: Differential expression and methylation analysis as a result of sample treatment with AZA (5-azacytidine) in MUC1.

**Additional file 8: Table S7** related to content in Fig. 4: Differential expression and methylation analysis as a result of sample treatment with AZA (5-azacytidine) in HUVEC.

**Additional file 9: Table S8** related to content in Fig. 4: Pathways enrichment analysis on genes exhibiting significantly lower promoter methylation and significantly higher expression upon 5 µM 5-azacytidine treatment.

**Additional file 10: Table S9** related to content in Fig. 4: Short description of genes common between those induced by AZA through promoter demethylation and those repressed in hCIMP tumors through promoter methylation.

**Additional file 11: Table S10** related to content in Fig. 4: Pathways enrichment analysis on genes exhibiting significantly higher promoter methylation and significantly lower expression in the H295R than in the MUC1 cells.

**Additional file 12: Table S11** related to content in Methods: Demographic and clinical data of the ACC TCGA cohort.

**Additional file 13: Table S12** related to content in Methods: Demographic and clinical data of the ENSAT TCGA cohort.

## Data Availability

The authors declare that all data supporting the findings of this study are available within the article and its Supplemental Data or from the corresponding author upon request. Sequencing data originated from cell lines are deposited in the Gene Expression Omnibus repository with reference GSE145560.

## Abbreviations

ACC: Adrenocortical carcinoma
AZA: 5-Azacytidine
CIMP: CpG island methylator phenotype
lCIMP, iCIMP, hCIMP: Low, intermediate, or high CIMP
FDR: False discovery rate
GSEA: Gene Set Enrichment Analysis
HUVEC: The primary human umbilical vein endothelial cell line
ENSAT: The European Network for the Study of Adrenal Tumors
RRBS: Reduced representation bisulfite sequencing
TCGA: The Cancer Genome Atlas
