# Supplementary material for: DNA hypermethylation driven by DNMT1 and DNMT3A favors tumor immune escape contributing to the aggressiveness of adrenocortical carcinoma
Source: Clin Epigenetics. 2023 Aug 2;15:121. doi: 10.1186/s13148-023-01534-5 (PMC10394822; doi:10.1186/s13148-023-01534-5)
Supplement: Supplementary file 1 — Additional file 1: Fig. S1. related to content in Fig. 1: DNMT1 and DNMT3A expression levels are upregulated in high CIMP ACC and are associated with poor clinical outcome. (a) Expression levels of DNMT1, DNMT3A, DNMT3B, TET1, TET2 and TET3 in the ENSAT patients exhibiting lCIMP (n = 20), iCIMP (n = 14) and hCIMP (n = 6) (two-sided t test, ***p < 0.005). (b) Kaplan–Meier estimates of overall survival for ACC patients, as a function of DNMT1 or DNMT3A. Fig. S2 related to content of Fig. 2: DNMT1 and DNMT3A expression is associated with high proliferation. Correlation between DNMT1 and DNMT3A expression and cell proliferation in the ENSAT patients. hCIMP (n = 6) samples are represented in red, iCIMP (n = 14) in orange and lCIMP (n = 20) in blue. Patients for whom the CIMP status was not defined are represented in gray. Fig. S3 related to content of Table 1: Gene Set Enrichment Analysis on TCGA datasets comparing hCIMP and lCIMP samples. (a) Representative example of gene sets enriched in hCIMP. (b) Representative example of gene sets enriched in lCIMP. Fig. S4, related to content of Fig. 3: CIMP in ACC is characterized by lower abundance of tumor-infiltrating immune cells Relative abundance of tumor-infiltrating immune and non-immune stromal cell populations, computed using MCP-counter, in lCIMP (n = 20), iCIMP (n = 14) and hCIMP (n = 6) samples from the ENSAT dataset. Comparison of relative abundances of each population using Kruskal–Wallis test followed by Dunn's test with Benjamini–Hochberg corrections. Fig. S5 related to content of Fig. 4: Impact of demethylating agent on cell proliferation in ACC cell lines. Effect of a 6-day treatment with the DNA methylation inhibitor 5-azacytidine (AZA) at 1, 5, or 10 µM in the H295R (n = 4), MUC1 (n = 5) cell lines and the non-tumor HUVEC cell line (n = 5). Cell growth was estimated by neutral red proliferation assay. p values of the Kruskal–Wallis test and Dunn’s test for stochastic dominance are reported: *p value < 0.05; * [file 13148_2023_1534_MOESM1_ESM.pdf]

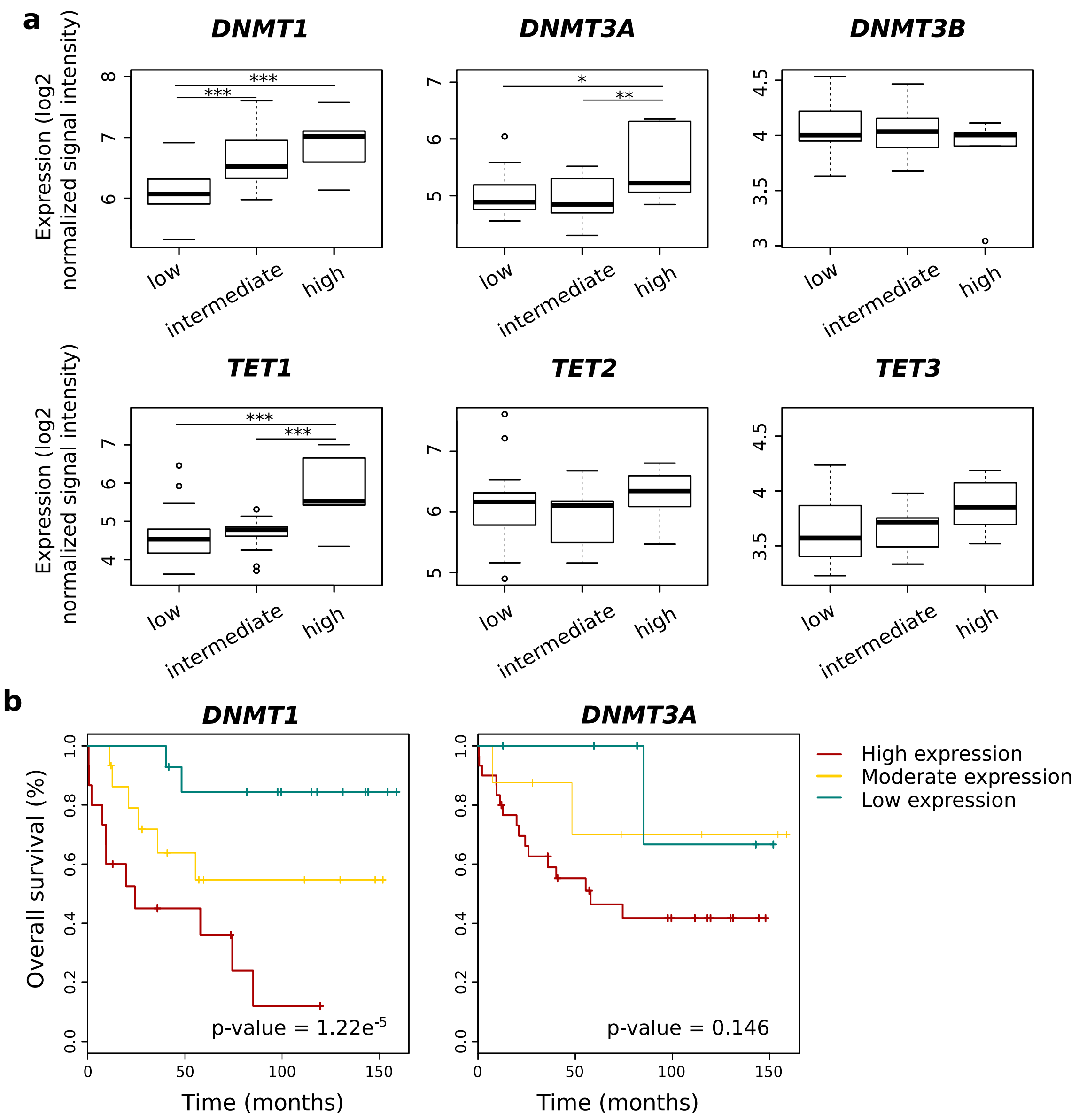


**Figure S1. *DNMT1* and *DNMT3A* expression levels are upregulated in high CIMP ACC and are associated with poor clinical outcome**

(**a**) Expression levels of *DNMT1*, *DNMT3A*, *DNMT3B*, *TET1*, *TET2* and *TET3* in the ENSAT patients exhibiting lCIMP, iCIMP and hCIMP (two-sided t-test, *p < 0.05, **p < 0.01, ***p < 0.005).

(**b**) Kaplan-Meier estimates of overall survival for ACC patients, as a function of DNMT1 or DNMT3A.

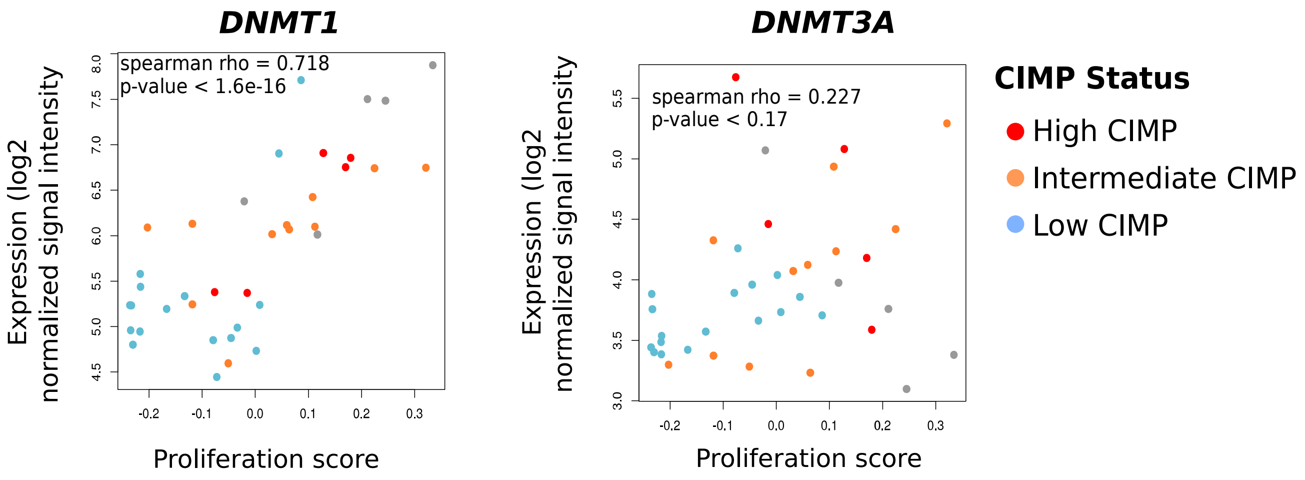


**Figure S2.** ***DNMT1*** **and** ***DNMT3A*** **expression is are associated with high proliferation**

Correlation between *DNMT1* and *DNMT3A* expression and cell proliferation in the ENSAT patients. hCIMP samples are represented in red, iCIMP in orange and lCIMP in blue. Patients for whom the CIMP status was not defined are represented in gray.

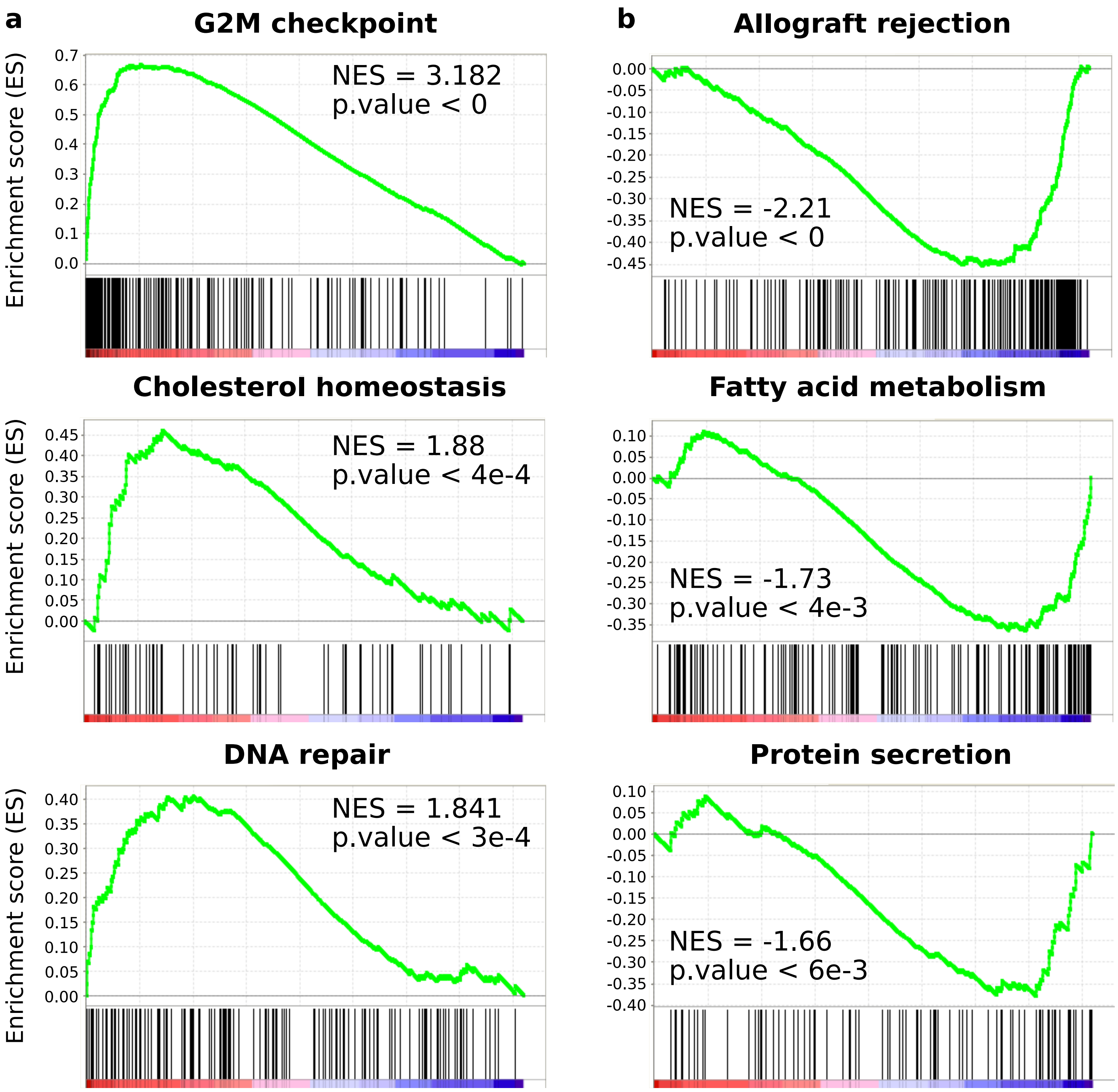


**Figure S3. Gene Set Enrichment Analysis on TCGA datasets comparing hCIMP and lCIMP samples**

(**a**) Representative example of gene sets enriched in hCIMP.

(**b**) Representative example of gene sets enriched in lCIMP.

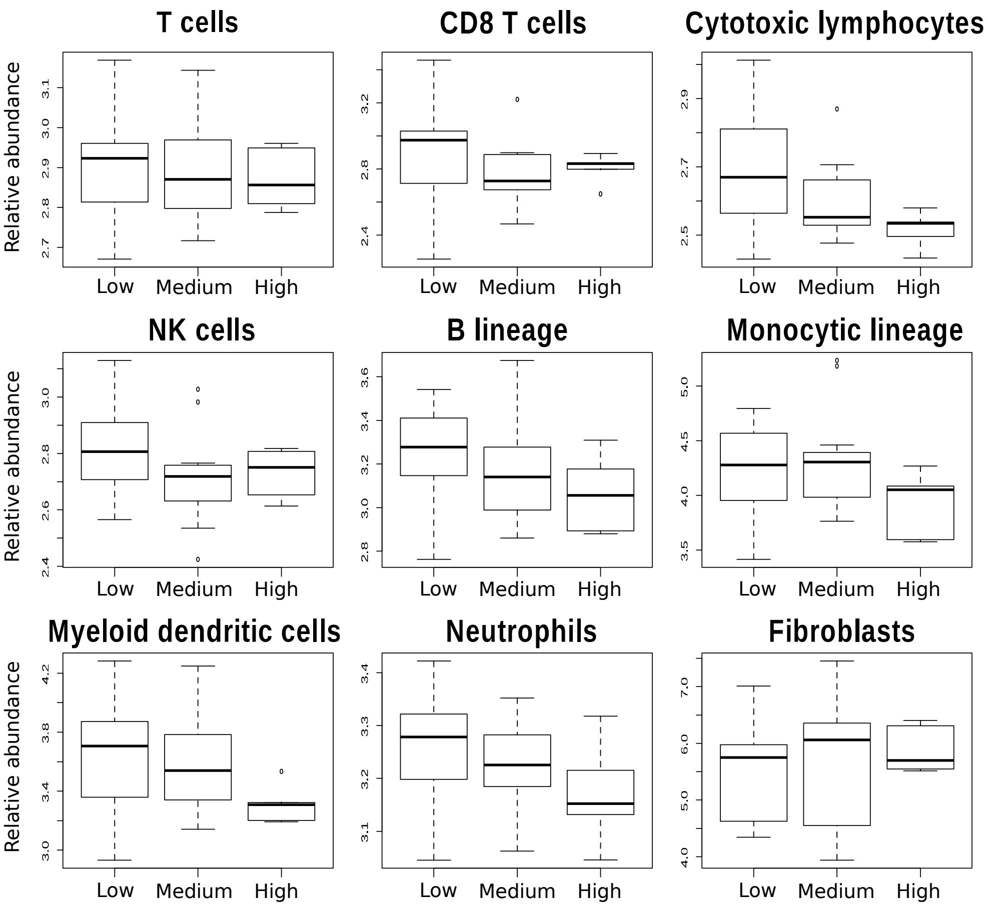


**Figure S4. CIMP in ACC is characterized by lower abundance of tumor-infiltrating immune cells**
Relative abundance of tumor-infiltrating immune and non-immune stromal cell populations, computed using MCP-counter, in lCIMP, iCIMP and lCIMP samples from the ENSAT dataset. Comparison of relative abundances of each population using Kruskal–Wallis test followed by Dunn's test with Benjamini–Hochberg corrections.

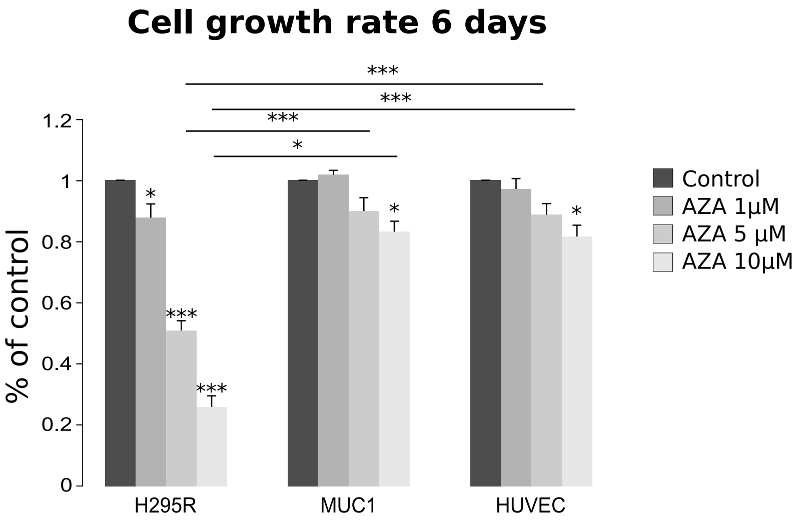


**Figure S5. Impact of demethylating agent on cell proliferation in ACC cell lines**

Effect of a 6-day treatment with the DNA methylation inhibitor 5-azacytidine (AZA) at 1, 5, or 10µM in the H295R (n=4), MUC1 (n=5) cell lines and the non-tumor HUVEC cell line (n=5). Cell growth was estimated by neutral red proliferation assay. *P*-values of the Kruskal–Wallis test and Dunn's test for stochastic dominance are reported: * $p$-value < 0.05; ** $p$-value < 0.01; *** $p$-value < 0.005

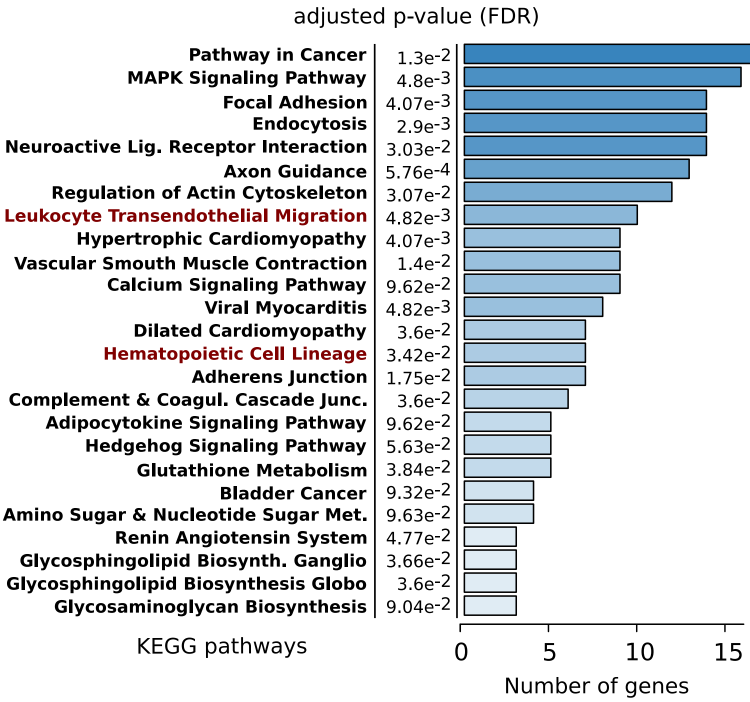


**Figure S6. Gene pathways differentially active in H295R compared to MUC-1 cells due to DNA hypermethylation**

Pathway enrichment analysis on genes that exhibit a significantly hypermethylated DMR in their promoter and significantly lower expression in H295R than in MUC-1 cells. Pathways related to the immune response are highlighted in red.
